# Randomised phase II study of ASA404 combined with carboplatin and paclitaxel in previously untreated advanced non-small cell lung cancer

**DOI:** 10.1038/sj.bjc.6604808

**Published:** 2008-12-09

**Authors:** M J McKeage, J Von Pawel, M Reck, M B Jameson, M A Rosenthal, R Sullivan, D Gibbs, P N Mainwaring, M Serke, J-J Lafitte, C Chouaid, L Freitag, E Quoix

**Affiliations:** 1 University of Auckland, 85 Park Road, Grafton, Private Bag 92019, Auckland 1142, New Zealand; 2 Asklepios Fachkliniken, Robert-Koch-Allee 2, D-82131 Gauting, Germany; 3 Krankenhaus Grosshansdorf, Woehrendamm 80, D-22927 Grosshansdorf, Germany; 4 Waikato Hospital, Private Bag 3200, Hamilton, New Zealand; 5 Royal Melbourne Hospital, Melbourne 3050, Victoria, Australia; 6 Auckland City Hospital, 2 Park Road, Grafton, Auckland 1023, New Zealand; 7 Christchurch Hospital, Private Bag 4710, Christchurch, New Zealand; 8 Mater Health Services, Brisbane 4101, Queensland, Australia; 9 Helios Klinikum Emil von Behring, Lungenklinik Heckeshorn, Walterhöferstrasse 11, D-14165 Berlin, Germany; 10 Hôpital Calmette Service de Pneumologie, Boulevard du professeur Jules Leclercq, 59037 Lille, France; 11 Hôpital Saint Antoine, 184, rue du Faubourg, Saint Antoine, 75571 Paris Cedex, France; 12 Lungenklinik Hemer, Theo-Funccius-Strasse 1, D-58675 Hemer, Germany; 13 Service de Pneumologie, Hôpitaux Universitaires, 67091 Strasbourg Cedex, France

**Keywords:** ASA404, AS1404, DMXAA, VDA, tumour-VDA, non-small cell lung cancer

## Abstract

ASA404 (5,6-dimethylxanthenone-4-acetic acid or DMXAA) is a small-molecule tumour-vascular disrupting agent (Tumour-VDA). This randomised phase II study evaluated ASA404 plus standard therapy of carboplatin and paclitaxel in patients with histologically confirmed stage IIIb or IV non-small cell lung cancer (NSCLC) not previously treated with chemotherapy. Patients were randomised to receive ⩽6 cycles of carboplatin area under the plasma concentration–time curve 6 mg ml^−1^ min and paclitaxel 175 mg m^−2^ (CP, *n*=36) or standard therapy plus ASA404 1200 mg m^−2^ (ASA404-CP, *n*=37). There was little change in the systemic exposure of either total or free carboplatin or paclitaxel on addition of ASA404. Safety profiles were similar and manageable in both groups, with most adverse effects attributed to standard therapy. Tumour response rate (31 *vs* 22%), median time to tumour progression (5.4 *vs* 4.4 months) and median survival (14.0 *vs* 8.8 months, hazard ratio 0.73, 95% CI 0.39, 1.38) were improved in the ASA404 combination group compared with the standard therapy group. In conclusion, this study establishes the feasibility of combining ASA404 with carboplatin and paclitaxel in patients with previously untreated, advanced NSCLC, demonstrating a manageable safety profile and lack of adverse pharmacokinetic interactions. The results indicate that there may be a benefit associated with ASA404, but this needs to be evaluated in a larger trial.

The mainstay of treatment for advanced non-small cell lung cancer (NSCLC) is platinum-based chemotherapy (Non-Small Cell Lung Cancer Collaborative Group, 1995), often combined with paclitaxel (Langer *et al*, 1995). However, an efficacy plateau has been reached with chemotherapy; no regimen is clearly superior (Schiller *et al*, 2002), and adding a third cytotoxic agent increases toxicity without improving outcome (Wakelee and Belani, 2005).

A new treatment strategy under investigation involves targeting tumour vasculature with small-molecule vascular disrupting agents (VDAs), such as the tubulin-depolymerising combretastatin A-4-phosphate (C4AP) (Dowlati *et al*, 2002; Stevenson *et al*, 2003; Rustin *et al*, 2003b) and the microtubule-independent ASA404 (5,6-dimethylxanthenone-4-acetic acid (DMXAA)), or anti-angiogenic agents, such as the antibody bevacizumab. In a phase III study, the combination of carboplatin, paclitaxel and bevacizumab improved survival significantly compared with carboplatin and paclitaxel alone in patients with advanced NSCLC of non-squamous histology (Sandler *et al*, 2006).

The tumour-VDA, ASA404, induces apoptosis of tumour vascular endothelial cells and cytokine production, leading to tumour vascular collapse (Ching *et al*, 2002; Baguley, 2003; Tozer *et al*, 2005). In animal models, this culminates in extensive tumour necrosis predominantly within the tumour core (Zwi *et al*, 1994; Laws *et al*, 1995; Ching *et al*, 1999, 2004; Joseph *et al*, 1999). The therapeutic potential of ASA404 appears to lie in its combination with other treatments (Wilson *et al*, 1998; Murata *et al*, 2001). In animal models, ASA404 acted synergistically with chemotherapy (Pruijn *et al*, 1997; Siim *et al*, 2003; McKeage and Kelland, 2006); therapeutic gains were most striking with taxanes. Scheduling studies indicated that activity was optimised when ASA404 was administered shortly after chemotherapy (Siim *et al*, 2003).

In two phase I trials, 109 patients received ASA404 monotherapy at doses of 6–4900 mg m^−2^ weekly or every 3 weeks (Jameson *et al*, 2003; Rustin *et al*, 2003a). ASA404 did not cause myelosuppression and was generally well tolerated. Transient prolongation of heart rate-corrected cardiac QT interval (QTc) was seen at high doses (⩾2000 mg m^−2^). Transient, dose-dependent visual disturbances were also noted. Dose-limiting toxicities were rapidly reversible and included confusion, tremor, slurred speech, visual disturbance, anxiety, urinary incontinence and possible left ventricular failure. The maximum tolerated dose (MTD) was 3700 mg m^−2^. ASA404 produced two unconfirmed partial responses at 1100 and 1300 mg m^−2^, and 28 patients had a best response of stable disease. Dynamic contrast-enhanced magnetic resonance imaging showed reductions in tumour blood flow at sub-MTD doses (Galbraith *et al*, 2002). A third phase I study investigated the potential for cardiac and ophthalmic toxicity and found ASA404 doses of 1200 and 1800 mg m^−2^ to be well tolerated, with no significant effect on QTc interval or deterioration in ophthalmic variables (McKeage *et al*, 2006). Near-maximal levels of the tumour vascular damage biomarker 5-hydroxyindoleacetic acid (5-HIAA) were seen at these doses (Baguley *et al*, 1997), and ASA404 plasma concentrations were within the preclinical therapeutic range (McKeage *et al*, 1991).

We conducted this randomised phase II study to determine the feasibility of combining ASA404 1200 mg m^−2^ with carboplatin and paclitaxel, to examine the potential for pharmacokinetic interactions between components of this regimen and to evaluate its safety and efficacy in patients with previously untreated advanced NSCLC.

## Materials and methods

Men and women ⩾18 years with histologically confirmed, locally advanced or metastatic NSCLC (stage IIIb/IV, incurable by surgery/radiotherapy), with ⩾1 unidimensionally measurable lesion according to the Response Evaluation Criteria in Solid Tumors (RECIST) (Therasse *et al*, 2000) and no previous chemotherapy were eligible. Other requirements included Karnofsky performance status ⩾70%, life expectancy ⩾3 months and adequate haematological, renal and hepatic function.

Main exclusion criteria were major surgery/radiotherapy (unless palliative) ⩽4 weeks before enrolment, central nervous system metastases, small cell or mixed lung cancer, clinically significant cardiac arrhythmia or known QTc interval prolongation, severe or uncontrolled systemic disease, pregnancy, use of medication known to affect systemic serotonin levels or QTc interval ⩽2 weeks before ASA404 administration or an expected need for such treatment during the study.

Patients were recruited from 15 centres in New Zealand, Australia, Germany and France. The study was conducted according to the Declaration of Helsinki. Ethics committee approval and informed patient consent were obtained.

### Study design

This randomised, open-label, phase II study tested the addition of ASA404 1200 mg m^−2^ to a standard therapy regimen comprising carboplatin dosed to area under the plasma concentration–time curve (AUC) 6 mg ml^−1^ min plus paclitaxel at an FDA-approved dose of 175 mg m^−2^.

Patients were allocated to receive ASA404 1200 mg m^−2^ plus carboplatin and paclitaxel (ASA404-CP) or carboplatin and paclitaxel alone (CP). Randomisation was conducted centrally, with stratification for disease stage, performance status and histological type. On day 1 of each cycle, patients received paclitaxel as a 3-h IV infusion, then carboplatin as a 30-min IV infusion, then, if allocated, ASA404 as a 20-min IV infusion.

As this was the first experience of ASA404 in combination with carboplatin and paclitaxel in humans, a single patient received the combination with ASA404 at a lower dose of 600 mg m^−2^ (six cycles) to check safety before randomisation began. Then, the first six patients randomised to the ASA404 group were recruited according to early stopping rules to monitor safety before the study progressed further. A detailed pharmacokinetic (PK) evaluation of the ASA404-CP combination was undertaken in these first six patients to assess the potential for drug interaction between ASA404 and paclitaxel and carboplatin in combination. To facilitate this, the ASA404 regimen was modified, with the first cycle of treatment comprising CP, then up to five cycles of ASA404-CP, and finally, for patients still on study, a single cycle of ASA404 alone.

On the basis that no more than one dose-limiting toxicity (DLT), not clearly attributed to paclitaxel or carboplatin, was observed in the first six patients, enrolment continued until approximately 35 eligible patients were recruited in each of the ASA404-CP and CP groups. Treatment was given every 21 days for six cycles or until withdrawal, whichever was earlier. Within-patient dose modification of ASA404 or carboplatin, and crossover between the ASA404-CP and CP arms were not permitted. The dose of paclitaxel remained unaltered unless dose reduction was required due to toxicity attributed to these agents.

### Assessments

Tumours were measured using computed tomography or magnetic resonance imaging scans. Tumour response was evaluated using RECIST and categorised as complete (CR), partial (PR), stable disease (SD) or progressive disease (PD). Response was confirmed by examination ⩾4 weeks after first assessment. An independent outcomes committee carried out a blinded radiological review of all tumour assessments.

Safety assessments included a symptom-directed clinical examination before each cycle, 8 and 15 days after drug administration (first six patients only) and at the safety follow-up visit. Laboratory safety assessments of haematology and biochemistry parameters were conducted at these same time points.

Intensive electrocardiographic assessments were conducted throughout the study. Three standard 12-lead ECGs were acquired during the 30 min before paclitaxel administration (at cycles 1, 2 and 6 for the first six patients receiving ASA404; at cycles 1 and 6 for all other patients), and at the end of carboplatin infusion (at cycle 1 for the first six patients receiving ASA404). Single ECGs were collected immediately before administration of ASA404 then at 10, 20, 60 min and 1, 2 and 4 h from the start of the ASA404 infusion (at cycles 2 and 6 for the first six patients receiving ASA404; at cycles 1 and 6 for all other patients). Electrocardiograms were also collected immediately before paclitaxel infusion and ASA404 infusion, and 1 h from the start of infusion (at cycles 3, 4 and 5 for the first six patients receiving ASA404; at cycles 2–5 for all other patients).

If a patient developed a QTc interval (Bazett's correction) >520 ms (men) or >540 ms (women), further ECGs were acquired until the QTc interval returned to within 30 ms of baseline on two consecutive ECGs.

In patients receiving ASA404, ophthalmic tests were performed before treatment and at follow-up. These included best-corrected visual acuity, ophthalmological examination, contrast sensitivity, colour vision/colour contrast sensitivity and central visual field.

For the first six patients recruited according to early stopping rules, serial PK samples were collected and analysed after carboplatin and paclitaxel dosing at cycle 1, after ASA404, carboplatin and paclitaxel dosing at cycle 2, and after ASA404 monotherapy, 3 weeks after the end of cycle 6. Total and free concentrations for each drug were determined before infusion, at the end of infusion and at various intervals following the end of infusion.

In addition, plasma samples were analysed for 5-HIAA concentration at 2 and 4 h following the start of ASA404 dosing on day 1 of cycle 2.

All patients attended a screening visit ⩽28 days before treatment, a study visit every week and a follow-up visit 4 weeks after study completion/withdrawal. Tumours were measured every 6 weeks until disease progression. Survival was assessed every 3 months.

### Statistical methodology and analysis

Study populations were defined prospectively. Eligible patients met the inclusion criteria and received ⩾1 dose of study treatment. An intention-to-treat (ITT) population (all patients allocated to treatment) was not prospectively defined.

According to the protocol, efficacy analyses were performed on all eligible patients who received ASA404-CP or CP and safety analyses were performed on all patients who received ⩾1 dose of study treatment. The single patient who received ASA404 at a dose of 600 mg m^−2^ was excluded from the analysis of safety and efficacy.

Primary safety outcomes were treatment-emergent adverse events (AEs), laboratory abnormalities, effect on QTc interval and ophthalmic toxicity. A treatment-emergent AE was defined as any unfavourable and unintended sign, symptom or disease temporally associated with the use of a medicinal product (including abnormal laboratory findings or worsening of pre-existing conditions), whether or not considered related to the study drug.

Adverse events were listed using MedDRA coding and graded according to the National Cancer Institute Common Terminology Criteria for Adverse Events (NCI-CTCAE) version 3.0, or as mild, moderate or severe if NCI-CTCAE were not applicable. Relationships of AEs to treatment were assessed as definite, probable, possible or unrelated.

Plasma concentrations of ASA404, carboplatin and paclitaxel were summarised at each time point, for each ASA404 dose and treatment cycle. Mean maximum observed concentration (*C*_max_) and AUC from time of dosing to time of last observation (AUC_(0–t)_) were calculated and expressed as a ratio (co-administration/alone).

Principal efficacy end points were objective response rates, time to tumour progression (TTP) and survival. According to the protocol, TTP and survival were defined as time from treatment initiation to first objective documentation of progression or death, respectively. In the absence of progression (TTP end point) or death (survival end point), data were ‘censored’ at the last follow-up date. Kaplan–Meier curves were fitted for TTP and survival and used to estimate median and 1-year values.

Treatment differences between the ASA404 plus CP and CP-alone groups were assessed by calculating the percentage difference (for response rates) and the hazard ratio (for time to event end points) with the corresponding 95% confidence interval and *P*-value. Statistically significant differences are indicated by *P*<0.05.

A total sample size of 70 patients was fixed. Assuming an overall response rate of 23% in the ASA404-CP arm, then the lower limit of the 95% confidence interval would be at least 10%.

## Results

### Patient demographics and disposition

Of the 76 patients allocated to treatment, three were excluded from the safety population as they received no study medication (ASA404-CP, *n*=2; CP, *n*=1); the safety population therefore comprised 73 patients (ASA404-CP, *n*=37; CP, *n*=36). Three treated patients from the ASA404-CP group were excluded from the eligible population because they did not meet inclusion criteria (*n*=1) or withdrew before receiving ASA404 (*n*=2); the eligible population therefore comprised 70 patients (ASA404-CP, *n*=34, CP, *n*=36).

The groups were well balanced for pretreatment characteristics (Table 1). Approximately one-third of patients had squamous cell carcinoma.

### Treatment

The average number of chemotherapy cycles was higher in the ASA404-CP group than in the CP group (4.3 and 3.8 cycles per patient, respectively). Eleven patients in the ASA404-CP group and seven in the CP group required paclitaxel dose reduction. Cycles were delayed more frequently in the ASA404-CP group than in the CP group (17.6 and 7.4% of all cycles, respectively).

### Pharmacokinetics

Pharmacokinetic evaluation was performed for the first six patients with the objective of assessing the potential for drug interaction between ASA404 and paclitaxel and carboplatin.

On cycle 1 for these six patients, the CP combination was given alone and PK parameters were found to be consistent with published values (Patnaik *et al*, 2006). On cycle 2, CP was co-administered with ASA404 and the PK parameters were compared with CP alone. Mean PK parameters for carboplatin and paclitaxel on cycle 2, expressed as ratio of those on cycle 1, are shown in Table 2. Co-administration of ASA404 did not fundamentally alter the PK parameters of either carboplatin or paclitaxel.

On cycle 7, ASA404 was administered alone and PK parameters were consistent with those reported previously from phase I studies (McKeage *et al*, 2006) and with those when co-administered with CP in this study. Mean PK parameters for ASA404 on cycle 2, expressed as a ratio of those on cycle 7, are shown in Table 2. Co-administration did not alter the mean systemic exposure to total ASA404; however, the *C*_max_ and AUC values for free ASA404 were increased approximately six- and eight-fold, respectively. Systemic exposure was markedly higher for total ASA404 compared with free ASA404, as expected for an agent that is extensively protein bound (Jameson *et al*, 2003).

### Pharmacodynamics

Peak levels of 5-HIAA occurred 2 h after ASA404 dosing. Mean concentrations (*n*=5) at this time point were 137.2±46.6 nM, representing an increase of 80.6±26.0 nM from baseline. This relative increase is similar to that seen with ASA404 1200 mg m^−2^ in phase I studies (Rustin *et al*, 2003a; McKeage *et al*, 2006).

### Safety

Overall safety profiles were generally similar in the ASA404-CP and CP groups (Table 3), and for squamous and non-squamous patients. Most AEs and serious AEs (SAEs) were attributed to standard therapy components. The proportions of patients with AEs, ASA404- or CP-related AEs, SAEs and deaths, or study discontinuations due to AEs, were similar in the two groups.

The most frequently occurring AEs attributed to ASA404 were infusion site pain, nausea, vomiting and anaemia. The only SAE directly attributed to ASA404 was asthenia in one patient.

The most common grade 3/4 toxicities are shown in Table 4. Grade 3/4 neutropenia, thrombocytopenia, infection, hypokalemia and infusion site burning or pain occurred more frequently in the ASA404-CP group, whereas grade 3/4 hyperglycaemia, neuropathy and anaemia occurred more frequently in the CP group.

Other differences noted between the two groups were four grade 3 respiratory AEs (dyspnea (*n*=2), pneumothorax and productive cough) occurring only in the ASA404-CP group and four grade 3 infections (chronic bronchitis, lung infection, urinary tract infection and viral diarrhoea) occurring in the ASA404-CP group *vs* one grade 3 infection (lower respiratory tract) occurring in the CP group.

Grade 3/4 haematological abnormalities occurred in 78.4 and 63.9% patients in the ASA404-CP and CP groups, respectively. The incidence of neutropenia at grade 3/4 was higher in the ASA404-CP group (62.2%) than in the CP group (38.9%) (difference of 23.3%, 95% CI 1.0, 45.6). Maximal decreases in neutrophil counts occurred at day 15 after cycle 6 in both groups. Mean absolute neutrophil count fell from 7.54±3.31 × 10^9^ l^−1^ at baseline to 1.14±1.0 × 10^9^ l^−1^ in the ASA404-CP group, and from 8.23±4.76 × 10^9^ l^−1^ to 1.54±0.75 × 10^9^ l^−1^ in the CP group. Laboratory data also showed that there was a higher overall incidence of thrombocytopenia (all grades) in the ASA404-CP group (62.2 *vs* 44.4%). Other haematological toxicities were similar in the two treatment arms.

The incidences of cardiac AEs and SAEs were higher in the ASA404-CP group than in the CP group (18.9 and 10.8% *vs* 8.3 and 2.8%, respectively). Four patients in the ASA404-CP group had cardiac SAEs. These were transient/reversible and included one instance each of tachyarrhythmia (cycle 1, grade 4), cardiomyopathy (cycle 3, patient withdrawn), myocardial ischaemia (cycles 3 and 5) and angina pectoris (cycle 3, grade 3). Three of these patients had a history of cardiovascular disease. Electrocardiogram analyses showed only one patient in the ASA404-CP group with a prolonged QTc interval.

No patient showed significant deterioration in ophthalmic variables after ASA404 treatment. Five AEs associated with visual function (irritation, blurred vision or visual disturbance) were seen in the ASA404-CP group and four were seen in the standard therapy group (blurred vision or visual disturbance). All visual function AEs were of <grade 3 severity.

Adverse events leading to withdrawal included disease progression (ASA404-CP, *n*=1; CP, *n*=3); anaphylactic reaction, paclitaxel reaction, hypersensitivity or premedication reaction (ASA404-CP, *n*=1; CP, *n*=3); leucopenia, neutropenia or thrombocytopenia (ASA404-CP, *n*=2; CP, *n*=1); peripheral neuropathy (CP, *n*=1); cardiomyopathy (ASA404-CP, *n*=1); and multiple chemotherapy toxicities (CP, *n*=1).

Seven deaths occurred on-study in the safety population (ASA404-CP, *n*=4; CP, *n*=3). These were attributed to disease progression (ASA404-CP, *n*=2; CP, *n*=1), pulmonary oedema (CP, *n*=1), pulmonary haemorrhage (CP, *n*=1), sepsis (ASA404-CP, *n*=1) and non-obvious reasons (ASA404-CP, *n*=1).

### Efficacy

Table 5 shows RECIST response outcomes. Investigator assessment gave a best overall response of PR, with 34.4% (95% CI 17.9, 50.8) and 29.0% (95% CI 13.1, 45.0) responses confirmed in the ASA404-CP group and CP group, respectively.

Independent assessment showed that a greater proportion of patients receiving ASA404 had a best overall response of PR with 31.3% (95% CI 15.2, 47.3) *vs* 22.2% (95% CI 6.5, 37.9) with CP, although it should be noted that 11 patients could not be evaluated for response.

**Figure 1:**
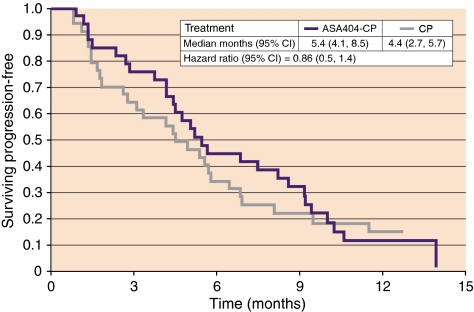
Kaplan–Meier estimate of the time to tumour progression; eligible population (ASA404-CP *n*=34, CP *n*=36).

Median TTP by investigator assessment was 5.4 months in the ASA404-CP group and 4.4 months in the CP group (Figure 1). The risk of progression was reduced by 14% in the ASA404-CP group, with a hazard ratio of 0.86, 95% CI 0.51, 1.45, and *P*=0.56 (for the ITT population: hazard ratio 0.94, 95% CI 0.56, 1.57, *P*=0.82).

**Figure 2:**
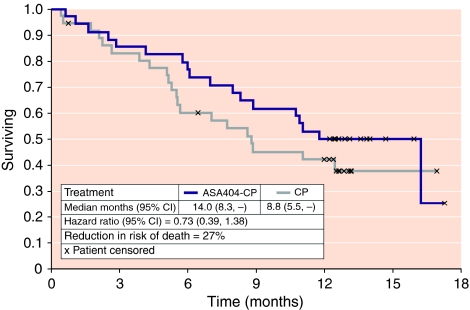
Kaplan–Meier estimate of the probability of survival; eligible population (ASA404-CP *n*=34, CP *n*=36).

Median survival was 14.0 months in the ASA404-CP group and 8.8 months in the CP group (Figure 2). The risk of death was reduced by 27% in the ASA404-CP group, with a hazard ratio of 0.73, 95% CI 0.39, 1.38, and *P*=0.33 (for the ITT population: hazard ratio 0.86, 95% CI 0.47, 1.57, *P*=0.63). One-year survival was 50.0% in the ASA404-CP group and 42.1% in the CP group.

## Discussion

This randomised phase II study evaluated the feasibility of adding the Tumour-VDA ASA404 to a standard regimen of carboplatin and paclitaxel in patients with previously untreated, advanced NSCLC.

The study showed that when ASA404 was co-administered with carboplatin and paclitaxel, there was little change in the systemic exposure or disposition of either total or free carboplatin or paclitaxel/6-alpha-hydroxy paclitaxel. Similarly, co-administration with this standard therapy did not markedly alter the systemic exposure of total ASA404. However, the concentration of free ASA404 was increased, suggesting that the chemotherapy drugs or excipients altered the partitioning of ASA404 within plasma.

The addition of ASA404 to carboplatin and paclitaxel was generally well tolerated and did not produce prohibitive additional toxicity. The ASA404-CP group had a similar overall safety profile to the CP group in that the incidences of AEs, SAEs and deaths or study discontinuations due to AEs were similar. The higher incidences of neutropenia, thrombocytopenia and infection in the ASA404-CP group compared with the CP group were not expected on the basis of the safety profile for ASA404 monotherapy (Jameson *et al*, 2003; Rustin *et al*, 2003a; McKeage *et al*, 2006). However, these were generally manageable and acceptable.

Intensive assessments showed that ophthalmic AEs occurred with similar incidences in the ASA404-CP and CP groups. No patient showed clinically relevant deterioration in ophthalmological parameters after ASA404 treatment. This suggests that ASA404 1200 mg m^−2^ can be combined with carboplatin and paclitaxel without the potential for the ophthalmic AEs seen at higher monotherapy doses (Galbraith *et al*, 2002; McKeage *et al*, 2006).

The incidence of cardiac AEs and SAEs was higher in the ASA404-CP group than in the CP group, although a causal relationship to ASA404 was not established. It can be noted that most of the cardiac SAEs in the ASA404-CP group occurred in patients with known cardiovascular disease. Furthermore, in phase I studies of ASA404, the predominant cardiac AE was QTc interval prolongation (Jameson *et al*, 2003; McKeage *et al*, 2006), of which there was a low incidence in this study. Nonetheless, as cardiac toxicity could result from the mechanism of action of VDAs (Cooney *et al*, 2004), the cardiac safety profile of ASA404 should continue to be monitored in future studies.

Although the study was not powered to compare efficacy outcomes statistically, the ASA404 combination appeared to improve a range of efficacy end points compared with carboplatin and paclitaxel alone – most notably overall survival. Response rates and survival in the CP group were similar to those reported previously for a carboplatin and paclitaxel regimen in patients with advanced NSCLC (Schiller *et al*, 2002).

The magnitude of improvement in TTP was more modest than that seen for overall survival. One possible explanation is that radiological measurements and RECIST may not detect the antitumour effects exerted by ASA404 because these are predominantly at the tumour core.

In a phase II study, addition of bevacizumab to a carboplatin and paclitaxel regimen in the same setting as in our study was associated with fatal pulmonary haemorrhage in patients with squamous histology (Johnson *et al*, 2004). A more recent study of the addition of the anti-angiogenic multiple kinase inhibitor sorafenib to carboplatin and paclitaxel also indicated a higher mortality rate in sorafenib-treated patients with squamous NSCLC (Scagliotti *et al*, 2008). Despite approximately one-third of patients in our study having squamous histology, only one episode of major pulmonary haemorrhage was documented and this occurred in the CP group. Other vascular-related side effects associated with bevacizumab (hypertension, proteinuria and epistaxis) (Johnson *et al*, 2004) were not prominent in the ASA404-CP group.

In conclusion, this study establishes the feasibility of combining ASA404 with a standard chemotherapy regimen of carboplatin and paclitaxel in patients with previously untreated, advanced NSCLC. The manageable safety profile, lack of adverse pharmacokinetic interactions and apparent improvements in various efficacy parameters associated with the addition of ASA404 to carboplatin and paclitaxel support the initiation of a phase III trial of sufficient size to test this novel combination regimen with statistical power.

## Figures and Tables

**Table 1:** Baseline characteristics of randomised patients (safety population)

	**ASA404-CP (*n*=37)**	**CP (*n*=36)**
Men, *n* (%)	23 (62.2)	24 (66.7)
Women, *n* (%)	14 (37.8)	12 (33.3)
Age (years), mean±s.d.	59.4±8.91	61.0±10.76
		
*Histological subtype,* n (%)		
Squamous cell carcinoma/undifferentiated	11 (29.7)	11 (30.6)
Adenocarcinoma	25 (67.6)	22 (61.1)
Large cell carcinoma	0	2 (5.6)
Other	1 (2.7)	1 (2.8)
		
*Stage,* n (%)		
IIIb	11 (29.7)	13 (36.1)
IV	26 (70.3)	23 (63.9)
		
*Karnofsky performance status,* n (%)		
70	1 (2.7)	3 (8.3)
80	9 (24.3)	9 (25.0)
90	17 (45.9)	14 (38.9)
100	10 (27.0)	10 (27.8)

ASA404-CP=ASA404 combined with carboplatin and paclitaxel; CP=carboplatin and paclitaxel; s.d.=standard deviation.

**Table 2:** Ratios (co-administration/alone^a^) of mean PK parameters for CP cycle 2/cycle 1 and for ASA404 cycle 2/cycle 7 (*n*=6)

	**Total carboplatin^b^**	**Free carboplatin^b^**	**Paclitaxel^c^**	**Paclitaxel metabolite^c^**	**Total ASA404^d^**	**Free ASA404^d^**
AUC_(0−t)_	1.19	1.38	1.10	0.96	1.05	7.95
95% CI	0.97, 1.41	0.98, 1.77	0.93, 1.27	0.62, 1.29	0.46, 1.64	2.05, 13.84
*C*_max_	1.26	1.36	0.88	1.04	1.01	5.72
95% CI	0.76, 1.76	0.37, 2.35	0.69, 1.07	0.69, 1.40	0.50, 1.52	2.01, 9.43

a In cycle 1 CP was given alone, in cycle 2 CP was co-administered with ASA404 and in cycle 7 ASA404 was given alone.

b *t*=20.25 h for carboplatin.

c *t*=45 h for paclitaxel.

d *t*=43.75 h for ASA404.

**Table 3:** Summary of treatment-emergent adverse events (safety population)

	**No. of patients (%)**	
	**ASA404-CP (*n*=37)**	**CP (*n*=36)**
*⩾1 adverse event*	34 (91.9)	36 (100.0)
Related to ASA404	27 (73.0)	—
Related to standard therapy	28 (75.7)	31 (86.1)
Grade 1^a^/mild^b^	3 (8.1)	4 (11.1)
Grade 2^a^/moderate^b^	6 (16.2)	8 (22.2)
Grade 3^a^/severe^b^	17 (45.9)	22 (61.1)
Grade 4a,^b^	5 (13.5)	0
Grade 5a,^b^	3 (8.1)	2 (5.6)
		
*⩾1 serious adverse event*	16 (43.2)	17 (47.2)
Related to ASA404	1 (2.7)	0
Related to standard therapy	5 (13.5)	6 (16.7)
Adverse event leading to death	2 (5.4)	2 (5.6)
Adverse event leading to withdrawal	5 (13.5)	9 (25.0)

ASA404-CP=ASA404 combined with carboplatin and paclitaxel; CP=carboplatin and paclitaxel.

a National Cancer Institute Common Terminology Criteria for Adverse Events grading.

b Worst severity grade.

**Table 4:** Most common grade 3 and 4 toxicities (safety population)

	**No. of patients (%)**			
	**ASA404-CP (*n*=37)**		**CP (*n*=36)**	
**Toxicity**	**Grade 3**	**Grade 4**	**Grade 3**	**Grade 4**
Neutropenia	8 (21.6)	15 (40.5)	10 (27.8)	4 (11.1)
Leukopenia	5 (13.5)	5 (13.5)	9 (25.0)	1 (2.8)
Alopecia	7 (18.9)	1 (2.7)	10 (27.8)	0
Hyperglycaemia	5 (13.5)	0	9 (25.0)	0
Neuropathy	2 (5.4)	0	7 (19.4)	0
Anaemia	3 (8.1)	0	5 (13.9)	0
Thrombocytopenia	2 (5.4)	2 (5.4)	2 (5.6)	0
Gastrointestinal disorders	3 (8.1)	0	2 (5.6)	1 (2.8)
Infection	4 (10.8)	0	1 (2.8)	0
Arthralgia, back or extremity pain	2 (5.4)	0	3 (8.3)	0
Cardiac disorders	3 (8.1)	1 (2.7)	1 (2.8)	0
Hypokalaemia	3 (8.1)	0	0	0
Respiratory, thoracic and mediastinal	3 (8.1)	0	0	0
Infusion site burning/pain	2 (5.4)	0	0	0
Dehydration	1 (2.7)	0	1 (2.8)	0
Anaphylactic shock or hypersensitivity	0	0	2 (5.6)	0
Neoplasms benign, malignant and unspecified	0	0	2 (5.6)	0
Flushing or hypotension	0	0	2 (5.6)	0
Febrile neutropenia	0	1 (2.7)	1 (2.8)	0

ASA404-CP=ASA404 combined with carboplatin and paclitaxel; CP=carboplatin and paclitaxel.

**Table 5:** Tumour response rate (eligible population)

	**No. of patients (%)**	
	**ASA404-CP (*n*=34)**	**CP (*n*=36)**
*Investigator assessment*		
Number of patients available for assessment	32 (100.0)	31 (100.0)
Partial response (confirmed)	11 (34.4)	9 (29.0)
Partial response (unconfirmed)	2 (6.3)	3 (9.7)
Stable disease	14 (43.8)	10 (32.3)
Progressive disease	5 (15.6)	9 (29.0)
		
*Independent assessment*		
Number of patients available for assessment^a^	32 (100.0)	27 (100.0)
Partial response	10 (31.3)	6 (22.2)
Stable disease	21 (65.6)	19 (70.4)
Progressive disease	1 (3.1)	2 (7.4)

ASA404-CP=ASA404 combined with carboplatin and paclitaxel; CP=carboplatin and paclitaxel.

a Tumour response could not be evaluated in 11 eligible patients by independent assessment due to: patient death before second tumour assessment (*n*=3); patient withdrawal before second tumour assessment (*n*=3); disease progression after cycle 1 and no subsequent scans available (*n*=2); no target lesion present (*n*=2); or no baseline scan available (*n*=1).
